# Cost-effectiveness of a multidimensional post-discharge disease management program for heart failure patients—economic evaluation along a one-year observation period

**DOI:** 10.1007/s00392-024-02395-5

**Published:** 2024-02-14

**Authors:** T. Egelseer-Bruendl, B. Jahn, M. Arvandi, S. Puntscher, J. Santamaria, L. Brunelli, K. Weissenegger, B. Pfeifer, S. Neururer, C. Rissbacher, A. Huber, B. Fetz, C. Kleinheinz, R. Modre-Osprian, K. Kreiner, U. Siebert, G. Poelzl

**Affiliations:** 1 Clinical Division of Orthopaedics and Traumatology, Medical University of Innsbruck, Innsbruck, Austria; 2 Institute of Public Health, Medical Decision Making and Health Technology Assessment, Department of Public Health, Health Services Research and Health Technology Assessment, UMIT TIROL-University for Health Sciences and Technology, Hall in Tirol, Austria; 3 Department of Internal Medicine III, Cardiology & Angiology, Medical University of Innsbruck, Innsbruck, Austria; 4 Interdisciplinary Heart Failure Center Tirol, IHZ, Anichstraße 35, 6020 Innsbruck, Tyrol Austria; 5 Tyrolean Federal Institute for Integrated Care, Tirol Kliniken GmbH, Innsbruck, Austria; 6 Division for Digital Medicine and Telehealth, UMIT TIROL - Private University for Health Sciences and Health Technology, Hall (Tyrol), Austria; 7 State Hospital – University Hospital, Innsbruck, Austria; 8 Department of Health, Federal State of Tyrol, Innsbruck, Austria; 9 TELBIOMED Medizintechnik Und IT Service GmbH, Graz, Austria; 10 Center for Health & Bioresources, AIT Austrian Institute of Technology, Graz, Austria; 11 Program On Cardiovascular Research, Institute for Technology Assessment and Department of Radiology, Massachusetts General Hospital, Harvard Medical School, Boston, MA USA; 12 Center for Health Decision Science and Departments of Epidemiology and Health Policy & Management, Harvard T.H. Chan School of Public Health, Boston, MA USA

**Keywords:** Heart failure, Disease management program, Telemedicine, Transitional care, Cost-effectiveness

## Abstract

**Objective:**

This study aimed to assess the cost-effectiveness of the telemedically assisted post-discharge management program (DMP) HerzMobil Tirol (HMT) for heart failure (HF) patients in clinical practice in Austria.

**Methods:**

We conducted a cost-effectiveness analysis along a retrospective cohort study (2016–2019) of HMT with a propensity score matched cohort of 251 individuals in the HMT and 257 in the usual care (UC) group and a 1-year follow-up. We calculated the effectiveness (hospital-free survival, hospital-free life-years gained, and number of avoided rehospitalizations), costs (HMT, rehospitalizations), and the incremental cost-effectiveness ratio (ICER). We performed a nonparametric sensitivity analysis with bootstrap sampling and sensitivity analyses on costs of HF rehospitalizations and on costs per disease-related diagnosis (DRG) score for rehospitalizations.

**Results:**

Base-case analysis showed that HMT resulted in an average of 42 additional hospital-free days, 40 additional days alive, and 0.12 avoided hospitalizations per patient-year compared with UC during follow-up. The average HMT costs were EUR 1916 per person. Mean rehospitalization costs were EUR 5551 in HMT and EUR 6943 in UC. The ICER of HMT compared to UC was EUR 4773 per life-year gained outside the hospital. In a sensitivity analysis, HMT was cost-saving when “non-HF related costs” related to the DMP were replaced with average costs.

**Conclusions:**

The economic evaluation along the cohort study showed that the HerzMobil Tirol is very cost-effective compared to UC and cost-saving in a sensitivity analysis correcting for “non-HF related costs.” These findings promote a widespread adoption of telemedicine-assisted DMP for HF.

**Graphical abstract:**

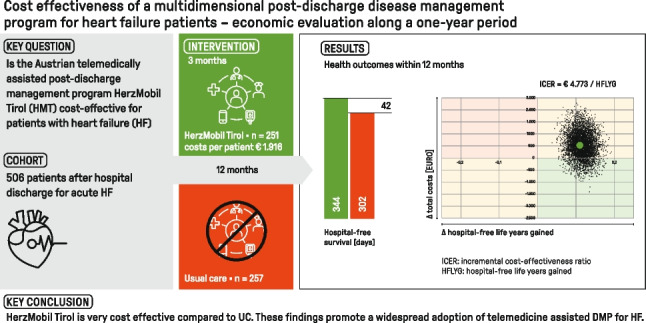

**Supplementary Information:**

The online version contains supplementary material available at 10.1007/s00392-024-02395-5.

## Introduction

Heart failure (HF) is recognized as an escalating public health problem in industrialized countries [1, 2]. Hospitalization for acute heart failure as the principal diagnosis is the most common cause of hospitalization in patients over 65 years of age [3]. In addition to a high 1-year mortality, such events are associated with frequent HF-related readmissions early after discharge [4], affecting approximately 25% of patients within the first 6 months [5].

Hospitalizations contribute significantly to increasing heart failure expenditures, accounting for an estimated 70% of total healthcare costs [6]. Prevention of hospital admissions is, therefore, of particular importance due to the health economic aspects. The cost-effectiveness of transitional care after heart failure hospitalization, including care in disease management clinics, nurse home visits, and nurse case management, has been shown compared with standard care [7]. This contrasts with studies of non-invasive telemedicine interventions, which so far have not shown clear cost-effectiveness [8, 9].

A recent health-economic evaluation of the Telemedical Interventional Management in Heart Failure II (TIM-HF2) trial in patients with a history of HF hospitalization within 12 months prior to randomization showed the cost-effectiveness of the intervention as compared to standard care alone [10]. However, the cost-effectiveness of a telemedical DMP in the vulnerable transition phase following an acute heart failure event has not yet been assessed. HerzMobil Tirol (HMT) is a multidimensional post-discharge disease management program (DMP), comprising a telemedical monitoring system incorporated in a comprehensive network of heart failure nurses and resident physicians and was effective in terms of reducing six-month HF-related readmissions and all-cause mortality [11].

The goal of this study was to evaluate the cost-effectiveness of the telemedical disease management program HMT for patients with advanced HF in the vulnerable phase following acute HF compared to standard of care along a 1-year cohort study.

## Methods

HMT is a 3-month transitional care disease management program for patients with HF that is established in clinical routine in the province of Tyrol, Austria. HMT uses a telemedical monitoring system that is integrated into a comprehensive network of healthcare providers. In a retrospective cohort study, which included hospitalized patients with decompensated heart failure regardless of the underlying left ventricular ejection fraction (LVEF), who required intravenous (iv) diuretics, the effects of the HMT were compared with usual care. Exclusion criteria were (1) multimorbidity (Charlson Comorbidity Index (CCI) > 6), (2) dementia, and (3) lack of willingness to participate.

### Study design and participants

The study type of our economic evaluation is an incremental cost-effectiveness study along a cohort study. Detailed methods on the cohort study are described elsewhere [11]. Briefly, propensity matching was applied to balance the compared arms for confounders. The age- and sex-matched control group (usual care–UC) with 257 individuals was based on the same inclusion criteria and recruited from patients admitted to hospitals from the same health-care provider and simultaneously as the study patients between 2016 and 2019.

In the HMT arm, 251 patients entered the program during hospitalization for acute HF (AHF). Patient education was delivered by specialized HF nurses. On discharge, each patient was assigned to a resident network physician near their home. Network physicians supervised the management of patients and gradually optimized evidence-based therapy. Each patient was provided an equipment set including a blood pressure and heart rate monitor and a weighing scale as well as a specially configured smartphone for daily data acquisition and transmission. Signal processing algorithms were used to analyze the transmitted physiological data and to identify upcoming adverse events. Automatic event detection indicated the need for immediate actions and fostered attention to those patients who needed early therapeutic interventions [12]. Face-to-face visits of patients with the network physician were scheduled 1, 4, and 12 weeks after discharge. HF nurses monitored patients’ compliance with medication, maintained telephone contact with patients if necessary, and adjusted HF medication according to the network physicians’ instructions. A home visit by the HF nurse was scheduled immediately after discharge to complete disease- and equipment-related education and to ensure that prescribed medication was available. At the end of the managed care program, the structured transfer of patients to usual care was organized.

Patients in UC underwent standard post-discharge planning, which typically included treatment plans and comprehensive discharge letters. In most cases, the actual follow-up of patients was unstructured and left to the respective family doctor or internist. Further details regarding the study design, intervention, data collection, and primary clinical results have been previously reported by Poelzl et al. [11].

### Health outcomes and effectiveness measures

The defined primary health outcome of the economic evaluation was hospital-free survival. Secondary outcomes included mean survival time and number and duration of rehospitalizations. All health outcomes were evaluated over the 1-year follow-up of the cohort study after inclusion of patients in HMT and UC. The effectiveness of HMT along the cohort study was calculated as absolute differences in health outcomes in the HMT versus the UC arm.

### Resource utilization and cost assessment

This economic evaluation along the 1-year cohort study was performed from the perspective of the Austrian health care system. We incorporated all high-cost resources as well as other relevant resources that are expected to differ between treatment arms [13] and, therefore, included direct costs of the DMP and costs of HF rehospitalizations. The costs of HMT include base costs (IT infrastructure, coordination, nursing staff), variable costs (license, helpdesk, support, fee for network physicians, laboratory costs), and costs for the equipment set including logistics. Salaries for doctors and nurses are also included in the ongoing basic costs, as well as costs for the necessary technical training for users. Base cost per patient case was calculated assuming a projected full expansion of HMT of 730 patients in Tyrol per year with an additional 10% of individuals with a 3-month extension summing up to 803 patient cases.

The costs for HF rehospitalizations were evaluated on the basis of the Diagnostic-Related Group (DRG) using the self-payer tariffs in Tyrol from 2022, which are based on a full cost calculation that includes variable costs and proportionate investment costs and hospital deficits. The state government applies these self-payer tariffs to calculate reimbursement for the hospitals, and therefore, these tariffs are typically used in economic evaluations. DRG points for the number of days in the hospital, use of individual medical service, and use of intensive care unit were multiplied by a factor of 1.363 EUR per DRG point, representing the average self-payer tariff weighted by the number of patients in each included hospital in Tyrol (Suppl. Table 1).

### Cost-effectiveness

We determined the cost-effectiveness of HMT vs. UC in terms of additional cost per hospital-free life-years gained (HFLYG) as our primary outcome. We first checked for dominance to identify strategies that provide less benefits at higher costs (dominated) and should therefore be eliminated or strategies that provide more benefits at lower costs (dominant) that should be given priority. If there was a trade-off between costs and health outcomes (not dominant or dominated), the incremental cost-effectiveness ratio (ICER), defined as additional costs (EUR) per HFLYG of HMT versus UC, was calculated. In addition, we calculated the incremental cost-effectiveness ratios expressed as additional costs (EUR) per HFLYG, per total life-years gained, and per number of rehospitalizations avoided of HMT vs. UC, respectively [14, 15].

### Base-case analysis

In the base-case analysis, all patients were included in the analysis along the 1-year trial period and HF-rehospitalization costs were winsorized.

### Sensitivity analyses

First, to make our analyses less dependent on random hospital events not related to heart failure, we performed a sensitivity analysis excluding the costs of HF rehospitalizations after events that were irrelevant for the stated research question. For this sensitivity analysis, we assumed that hospital stays that are not directly related to chronic HF (i.e., costs due to heart transplantation in two patients, implantation of an assist device in one patient, and elective cardiac surgery in one patient in the HMT group and prolonged ICU stays for sepsis-related renal failure in two patients in the UC group) did not depend on the strategy HMT or UC. In a first analysis, such non-HF-related costs for a total of six patients were replaced by the average costs in the respective group. In a second analysis, we removed the entire data of these six patients and assessed cost-effectiveness based on these updated data sets. Second, we performed a nonparametric sensitivity analysis with bootstrap sampling (*n* = 5000 replications) from the patient-level data as an empirical probability distribution to assess the uncertainty around cost-effectiveness. Results were plotted in a cost-effectiveness plane, and mean outcomes and confidence intervals are presented. Third, we performed a sensitivity analysis on the costs per DRG point.

### Statistical analysis

Data preparation included winsorizing, missing value imputation, and adjustment for remaining confounding due to imperfect matching. HF-rehospitalization data were evaluated following the intention-to-treat (ITT) principle. In the winsorization, costs above the 99th percentile were set to the 99th percentile. Missing values were replaced using imputation. First missing values of predictors for outcomes (i.e., body mass index (BMI), atrial fibrillation, N-terminal pro-B-type natriuretic peptide (NTproBNP), and CCI) were imputed by the mean values of the study arm with missing values assuming missingness completely at random (BMI missing = 4; atrial fibrillation missing = 1; NTproBNP missing = 20; CCI missing = 1). Second, missing values of the DRG points for hospital days, intensive care use, and individual medical services were imputed by the conditional means from a generalized linear model to fit a multivariable gamma regression with log-link function assuming a structure of missingness at random (MAR) (missing = 15 cases in the HMT arm). Variables in the regression to predict the DRG points are (1) study arm (exposure) and two potential predictors for the costs: sex, age, ejection fraction categories (reduced, mildly reduced, preserved), heart failure first diagnosed more than 18 months ago, CCI, atrial fibrillation, New York Heart Association (NYHA) functional class, NTproBNP, and cardiac resynchronization therapy (CRT) and/or implantable cardioverter-defibrillator (ICD). Adjustment for the remaining confounders was performed by regression analysis of rehospitalization costs, using a generalized linear model to fit a multivariable gamma regression with log-link function, accounting for zero-cost observations in the imputed Winsorized data. Variables in the regression model [16] to predict total HF-rehospitalization costs are (1) study arm (exposure) and (2) potential predictors for the total costs: sex, age, ejection fraction categories (reduced, mildly reduced, preserved), heart failure first diagnosed more than 18 months ago, CCI, atrial fibrillation, NYHA functional class, NTproBNP, and CRT cardiac resynchronization therapy and/or ICD.

For health outcomes, between-group comparisons were performed with the Student’s *t*-test, Mann–Whitney U test, and Chi-square test. A two-sided* p*-value of 0.05 was considered to be statistically significant. HF-rehospitalization and mortality data were evaluated following the intention-to-treat (ITT) principle.

For the statistical analysis including the cost-effectiveness analysis, we used SAS for Windows, Ver. 9.4 (SAS Institute Inc., Cary, NC, USA) and MS Excel, Ver. 2208 (Microsoft Corp, Redmond, USA).

We followed international guidelines for performing observational real-world studies [17, 18], reporting cost-effectiveness analyses such as the ISPOR Good Research Practices Task Force Report on Cost-effectiveness Analysis alongside Clinical Trials II [13] and the Consolidated Health Economic Evaluation Reporting Standards (CHEERS) Statement [19, 20].

## Results

Demographic and clinical characteristics of the population are shown in Table 1 and in Poelzl et al. [11]. Briefly, mean age was 69.5 years (standard deviation (SD) ± 11.9 years) in HMT and 71.1 years (SD ± 10.8 years) in UC, with 75 (29.9%) female patients in HMT and 83 (32.3%) in UC and a BMI of 28.3 kg/m^2^ (SD ± 5.7 kg/m^2^) in HMT and 27.3 kg/m^2^ (SD ± 5.6 kg/m^2^) in UC.

**Table 1:** Patient characteristics at baseline

Variable	Usual care (*n* = 257)	HerzMobil Tirol (*n* = 251)	*p*-value
Age, years (mean ± SD)	71.1 ± 10.8	69.5 ± 11.9	0.151^+^
Female, *n* (%)	83 (32.3%)	75 (29.9%)	0.557^++^
Body mass index, kg/m2, (mean ± SD)	27.3 ± 5.6	28.3 ± 5.8	0.029^+^
NYHA functional class, *n* (%)			0.180^++^
II	83 (32.3)	66 (26.3)	
III	168 (65.4)	182 (72.5)	
IV	6 (2.3)	3 (1.2)	
NTproBNP (ng/l), (mean ± SD)	3486 (1459–7294)	2991 (1750–5459)	0.367^+++^
HF first diagnosed > 18 months ago, n (%)	113 (44.0)	102 (40.6)	0.447^++^
Ejection fraction classification, *n* (%)			0.0003^++^
HFrEF (< 40%)	109 (42.4)	149 (59.6)	
HFmrEF (40–50%)	56 (21.8)	47 (18.8)	
HFpEF (> 50%)	92 (35.8)	55 (21.6)	
Atrial fibrillation, *n* (%)	104 (40.5)	131 (52.2)	0.027^++^
CCI (mean ± SD)	2.8 ± 1.6	2.8 ± 1.5	0.996^+^
CRT and/or ICD, *n* (%)	43 (16.7)	51 (20.3)	0.298^++^

^+^*t*-test; ^++^ Chi-square-test; ^+++^ Mann–Whitney *U* test

*SD*, standard deviation; *n*, number; *NYHA*, New York Heart Association; *HF*, heart failure; *HFrEF*, heart failure with reduced ejection fraction; *HFmrEF*, heart failure with mid-range ejection fraction; *HFpEF*, heart failure with preserved ejection fraction; *CCI*, Charlson Comorbidity Index; *NTproBNP*, N-terminal pro-B-type natriuretic peptide; *CRT*, cardiac resynchronization therapy; *ICD*, implantable cardioverter-defibrillator

### Health outcomes and effectiveness measures

Health outcomes and effectiveness measures of HMT vs. UC expressed as absolute differences are displayed in Table 2. One-year all-cause mortality was recorded in 25 (10.0%) vs. 66 (25.7%) patients and 1-year HF hospitalizations in 62 (24.7%) vs. 89 (34.6%) patients in HMT and UC. Days to death or first HF hospitalization were 216.0 vs. 150.7, and 122.3 vs. 113.5 for HMT and UC, respectively. HMT resulted in 41.88 additional hospital-free life days, 40.19 additional life days, and 0.12 avoided hospitalizations in comparison to UC.

**Table 2:** Health outcomes HerzMobil Tirol and usual care, per patient

Health outcomes within 1 year (per patient)	Usual care (*n* = 257)	HerzMobil Tirol (*n* = 251)	Difference (HMT-UC)
Hospital-free survival [days]	301.72	343.60	41.88
Survival days [days]	309.97	350.16	40.19
Number of HF rehospitalizations	0.59	0.47	-0.12

*HMT*, HerzMobil Tirol; *UC*, usual care; *HF*, heart failure

A more detailed representation of the differences in hospital-free survival, life-years, and number of hospital admissions within 1 year between HMT and UC is given in Supplementary Table 2.

Mean DMP costs for HMT were 1915.57 EUR per patient case participating in the program (Table 3). Mean rehospitalization costs during follow-up per patient were 5551 EUR in the HMT and 6943 EUR in the UC arm (Table 4).

**Table 3:** Costs of HerzMobil Tirol

DMP HerzMobil Tirol cost items	Costs [EUR]
Base costs*	
IT infrastructure, coordination, nursing staff—Total per year (= Total)	805,729
Base costs per patient case (= Total cost/patient cases**)	1003.40
Variable costs per patient case	505.00
License, helpdesk, support, network physicians, laboratory costs	
Equipment set including logistics per patient	356.67
DMP HerzMobil Tirol costs***	**1915.57**

*DMP*, disease management program; *EUR*, euro

* One-off costs for the software application are not considered

**Cases per year stabilization = 730, extensions per year (10%) = 73, total patient cases per year = 803

***HMT DMP costs = 1003.4 EUR (base costs) + 505 EUR (1 + 0.10) (variable costs per patient case) + 356.67 (equipment) = 1915.57 EUR

**Table 4:** Cost of rehospitalizations and total costs base-case analysis

Mean costs [EUR]	Usual care (*n* = 257)	HerzMobil Tirol (*n* = 251)	Difference (HMT-UC)
Mean HF-rehospitalization (w/o DMP) costs per patient	6943	5551	− 1392
Base-case total costs (including DMP intervention cost)	6943	7466	523
Mean costs per rehospitalization	8670 (*n* = 89)	8737 (*n* = 62)	67
Mean cost of hospitalization day per patient	561 (*n* = 89)	555 (*n* = 62)	− 6

*DMP*, disease management program; *EUR*, euro; *HMT*, HerzMobil Tirol; *HF*, heart failure; *UC*, usual care; *w/o*, without

DMP costs = 1916 euro

A more detailed representation of cost differences due to benefits in hospital-free survival, life-years gained, and fewer hospitalizations within 1 year between HMT and UC is provided in Supplementary Table 2.

### Cost-effectiveness

The estimated hospital-free life years and costs of the base-case analysis for patients who receive usual care or participate in HMT are shown in Table 5. Mean hospital-free life-years were determined to be 0.94 for HMT and 0.83 years for usual care resulting in a gain of 0.11 hospital-free life years for HMT. This gain in life years out of hospital came at the additional cost of 523 EUR yielding an ICER of 4773 EUR per HFLYG.

**Table 5:** Cost-effectiveness analysis HerzMobil Tirol vs. usual care—base case analysis

	Hospital-free survival [years]	Incremental hospital-free survival years	Total costs [EUR]	Incremental total costs [EUR]	ICER [EUR/HFLYG]
Usual care	0.83	0.11	6943	523	4773
HerzMobil Tirol	0.94		7466		

*EUR*, euro; *ICER*, incremental cost-effectiveness ratio; *HFLYG*, hospital-free life years gained

A more detailed representation of ICER due to benefits in hospital-free survival, life-years gained, and fewer hospitalizations within a year between HMT and UC is provided in Supplementary Table 2.

### Sensitivity analyses

In the sensitivity analysis excluding costs of HF rehospitalizations after events irrelevant for the stated research question (i.e., costs due to heart transplantation in two patients, implantation of an assist device in one patient, and elective cardiac surgery in one patient in the HMT group and prolonged ICU stays for sepsis-related renal failure in two patients in the UC group), HF-rehospitalization costs in HMT decreased to 3124 EUR and in usual care to 5995 EUR. Total costs for patients in HMT (5040 EUR) and usual care and life expectancy out of hospital are summarized in Table 6. In this sensitivity analysis, HMT is less costly and more effective compared to UC, that is, a dominant technology.

**Table 6:** Cost-effectiveness analysis HerzMobil Tirol vs. usual care in a sensitivity analysis excluding irrelevant hospitalization costs

	Event-free survival [years]	∆ Event-free survival years	Total costs [EUR]	∆Total costs [EUR]	ICER [EUR/HFLYG]
Usual care	0.83	0.11	5995	− 955	HMT Dominant*
HerzMobil Tirol	0.94		5040		

*Cost-saving; *ICER*, incremental cost-effectiveness ratio; *HFLYG*, hospital-free life years gained

**Fig. 1:**
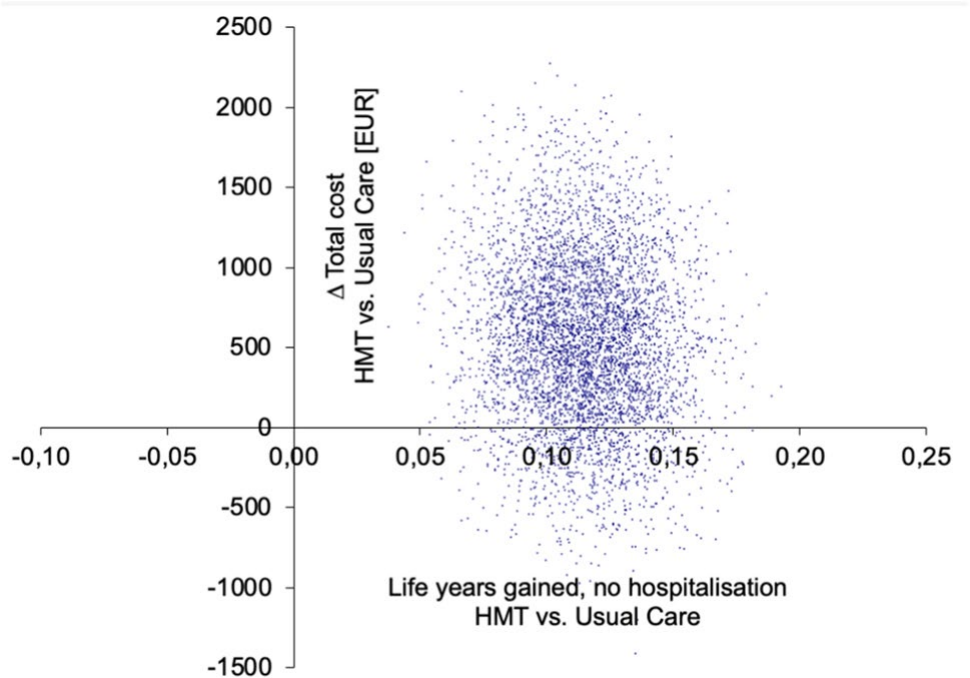
Cost-effectiveness plane HerzMobil Tirol vs. usual care for base case. The difference in hospital-free survival years gained is plotted on the x-axis, and the difference in total costs between HMT and UC is plotted on the y-axis. The point cloud shown is the result of bootstrap simulations. The majority of points in the base case analysis lie in the upper right quadrant, which represents a reasonable trade-off between higher cost and greater benefits. A total of 14.4% of the points are in the lower right quadrant, showing HMT as the dominant (i.e., less costly and more effective) therapy

**Fig. 2:**
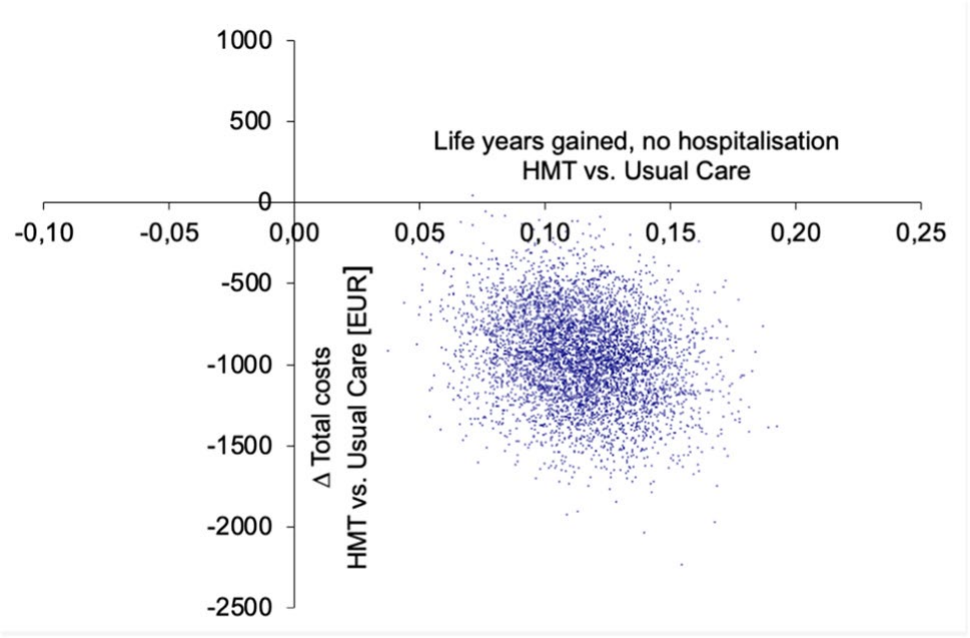
Cost-effectiveness plane HerzMobil Tirol vs. usual care for sensitivity analysis. The difference in hospital-free survival years gained is plotted on the x-axis, and the difference in total costs between HMT and UC is plotted on the y-axis. The point cloud shown is the result of bootstrap simulations. In the sensitivity analysis, all points are in the lower right quadrant, indicating that HMT is the dominant (i.e., less costly and more effective) therapy resulting in a cost saving of EUR 955 per patient

When the dataset of six patients with non-HF-related costs was completely removed, HMT was also less costly and more effective compared to UC (Table S3). The results of the nonparametric sensitivity analysis with bootstrap sampling (*n* = 5000 replications) plotted into a cost-effectiveness plane are displayed in Fig. 1 for the base-case analysis and in Fig. 2 for the sensitivity analysis with non-HF-related hospitalization costs replaced by mean costs. Bootstrapping results for the analysis where the dataset of six patients with non-HF-related costs was completely removed are provided in the supplement (Figure S1).

**Fig. 3:**
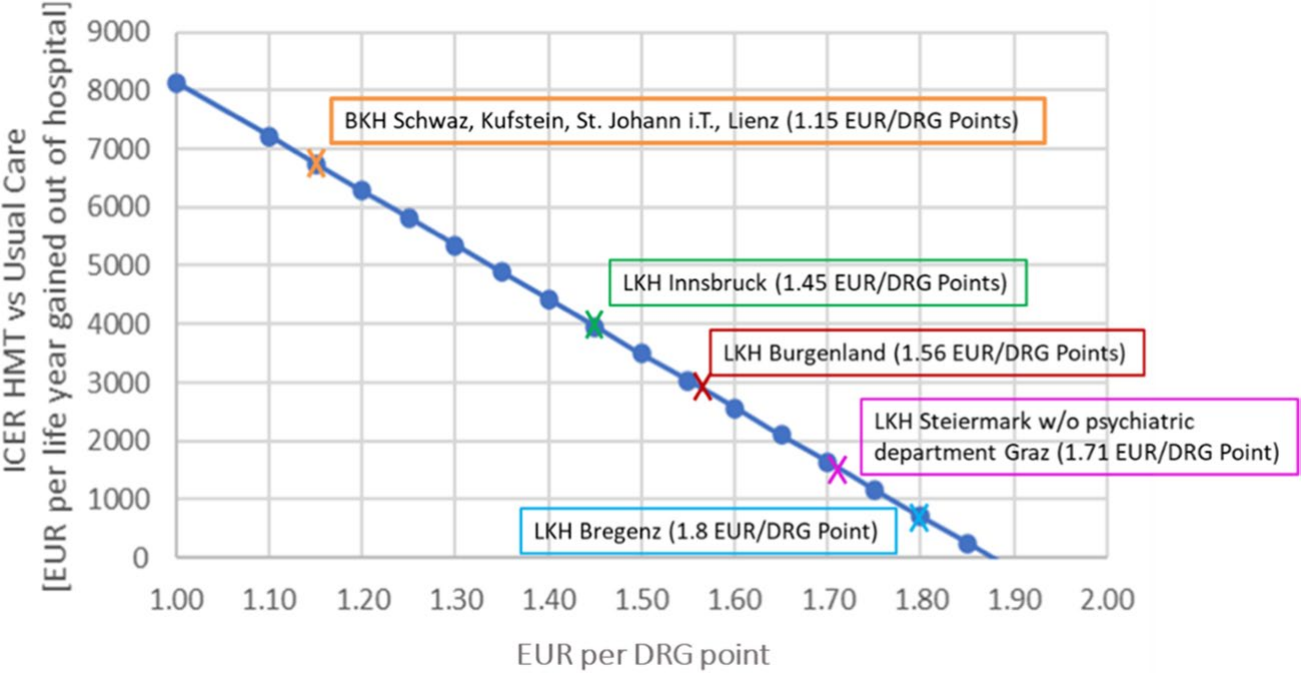
Cost-effectiveness in relation to DRG points. DRG points are the multiplier for the monetary costs per service in the respective hospitals in Austria. The ICERs are plotted against the DRG points. The crosses on the straight line mark the valid DRG points of the respective hospitals. The graph shows the increasing cost-effectiveness of HMT with increasing DRG points

The sensitivity analysis on the costs per DRG point is displayed in Fig. 3. With increasing costs per DRG point, the ICER decreases leading to further improved cost-effectiveness ratio for HMT.

## Discussion

To the best of our knowledge, this is the first cost-effectiveness study performed for a telemedical DMP for heart failure in the context of the Austrian health care system and the first at all in a real-world setting outside of a randomized controlled trial. The principal finding of this 1-year retrospective cohort study is that HerzMobil Tirol, a 3-month telemedicine-assisted transitional care service in patient with advanced heart failure, is highly cost-effective and improves health outcomes when compared with UC.

The present economic evaluation builds on data on the superior effectiveness of HMT, that is, reduction in heart failure hospitalization and mortality that were previously published [11]. The cost-effectiveness that largely offset the increased cost of the DMP occurred during follow-up as a result of a decrease in worsening HF and fewer deaths.

HMT resulted in an average of 42 additional hospital-free life days, 40 additional life days, and 0.12 avoided hospitalizations per person compared with usual care. The mean DPM costs for HMT were 1916 EUR per person. Hospital costs per day and patient were comparable between groups (€ 555 vs € 561). Mean HF-rehospitalization costs per patient were 5551 EUR in HMT and 6943 EUR in the UC arm. Total costs per person including DMP costs in HMT were on average 523 EUR higher than the costs in UC leading to an ICER of 4773 EUR per life-year gained outside the hospital.

Based on the results and bootstrap sampling, these results appear to be robust, showing that the HMT-based strategy was a more than acceptable cost-effectiveness trade-off in most bootstrap simulations and even dominant (cost savings, i.e., lower costs and better health outcomes) in 14.4% of simulations.

Sensitivity analyses showed a cost-saving effect of HMT when non-HF-related costs in six patients were replaced by the average costs in the HMT and UC groups and when these patients with non-HF-related hospital costs were completely removed from analyses. No random sample has shown that HMT is dominated by UC, i.e. more expensive and less effective.

The fact that HMT is cost-effective in the conservative base-case analysis and even cost-saving in the sensitivity analysis emphasizes the usefulness of DMPs, also from the perspective of the payers and the impact on the healthcare budget.

In Austria, there is no explicit threshold for an intervention to be considered cost-effective. In the international context, for example, in the USA and the UK, thresholds ranging from 50,000 USD per QALY gained to 150,000 USD per QALY gained and above have been reported [21, 22]. HMT can thus be considered as very cost-effective compared to usual care.

In general, telemedicine interventions in HF are relatively heterogenous, making a comparison difficult [9, 23]. Structured telephone support and telemonitoring for HF patients were found to reduce all-cause mortality and HF-related hospitalization [24–27]. Although only a few studies reported cost savings from economic evaluation as their primary aim [10, 28], most of the reports point in the same direction of potentially cost savings. In these studies, savings resulted almost entirely from reductions in hospital admissions, while other costs were comparable in intervention and control groups.

TIM-HF2, the largest trial to date with a comprehensive cost-effectiveness analysis demonstrating superior clinical effectiveness as well as relevant cost savings, is not conceptually exactly comparable to HMT, but nevertheless represents an important benchmark [10]. In the TIM-HF2 study, which did not include patients in the vulnerable transition phase, noninvasive remote patient management (RPM) was compared with usual care. The study included an HF patient education program followed by monthly patient telephone interviews with a study duration of 12 months and a follow-up period of up to 393 days after study onset. The RPM in TIM-HF2 [10] was cost-saving with average health-care costs per patient year of 14,412 EUR in the RPM group and 17,537 EUR in the UC group based on 339.08 days alive and out of hospital in the RPM versus 332.25 in the usual care group. The improved health outcome of 6 days alive and out of hospital was lower compared to 42 days gained in HMT. The total costs in TIM-HF2 included additional cost items such as non-HF-hospital stays, medication, and rehabilitation, and they are, therefore, considerably higher compared with HMT. The difference of unplanned HF hospitalizations in RPM versus usual care was − 522.65 EUR and for unplanned cardiovascular hospitalizations − 4,520.21 EUR.

In contrast to TIM-HF2, where the team was available 24/7 for 12 months to review RPM data, in HMT, comprehensive care by network physicians and HF nurses was provided only during office hours and for 3 months. Despite the longer supervision in TIM-HF2, the intervention costs in TIM-HF2 were 503 EUR less than in our study (RPM 1,414 EUR, HMT 1,916 EUR). This was mostly due to 25% higher personnel costs for running HMT compared to TIM-HF2. In contrast to TIM-HF2, HMT included home visits by heart failure nurses and a flat rate for the resident physicians. The relative costs for technical infrastructure (150 EUR [8%] vs. 85 EUR [6%]) and for patients measuring devices (357 EUR [19%] vs 226 E’UR [16%]) were higher in HMT where equipment logistics and reprocessing were included in the HMT equipment costs.

Taking all these differences into account, an ICER of € 4773 calculated in HMT is quite comparable to the cost savings of € 1758 per patient year in TIM-HF2.

### Strengths and limitations

HerzMobil Tirol is established in routine healthcare for advanced HF in the province of Tyrol, Austria. To the best of our knowledge, the present study is the first economic evaluation of a telemedicine-based transitional care program outside of a clinical study, meaning that “all-comers” rather than carefully selected study participants in a controlled setting were included. The present results demonstrate that the cost-effectiveness data collected in clinical trials are replicable in routine clinical practice.

However, as all empirical real-world economic evaluations, our study has several limitations.

First, the analysis only considered a 1-year time horizon of the clinical follow-up and may, therefore, reflect only partial (short-term) clinical and economic benefits of the DMP program [29]. Results may further improve with long-term effectiveness results of HMT. Such an analysis would require decision-analytic modeling using further assumptions [30].

Second, we did only partly apply weighted end points to synthesize information on quality and quantity of life by calculating hospital-free survival. We did not use utilities to calculate quality-adjusted life years (QALY) due to limited information on disease-specific quality of life or utility measures. However, in TIM-HF2, outcomes related to survival days and hospitalizations were consistent with QALY outcomes [11]. We opted for the partially weighted long-term outcome of hospital-free survival and did not focus on further time to event analyses as the primary outcome, as there are also substantial costs after the first event. However, from a clinical perspective, such analyses could be performed in the future.

Third, nonmedical resources (e.g., transportation to the hospital, patient time) were not considered. This likely biased our results against HMT because the inclusion of transportation costs or productivity loss for caring relatives would likely even decrease the incremental total costs of HMT in the base-case analysis. Also, productivity losses were not considered since most of the patients were already retired.

Fourth, in this retrospective analysis, resource use was not evaluated in the outpatient care setting. For simplification, it was assumed that medical treatment, rehabilitation, etc. were similar in both groups. In TIM-HF2, no substantial difference was found in medication and outpatient costs between groups. Rehabilitation costs were significantly higher in the DMP group for individuals who received rehabilitation, but there was no significant difference in the rehabilitation costs averaged for all individuals in both arms.

Fifth, the costs for the disease management program were based on projections of costs when HMT will be implemented across Tyrol. Due to the scalability of the base infrastructure (coordination and IT infrastructure), approximately 18% of the base costs per year will decrease with more patient cases managed year.

Sixth, since less than 5% of patients refused to participate in HMT, an overly optimistic assessment of the effectiveness of the program due to the exclusion of unstable or disinterested patients can largely be excluded. Inequalities in the baseline characteristics due to the retrospective nature of the study were balanced by multivariate testing and subgroup analyses [11].

Seventh, one-off costs for the software application were not taken into account, as HMT was already introduced in 2012. As these costs are very low at around €20,000 and are spread over all future patient years, they do not influence the cost-effectiveness in any relevant way, but must be taken into account when planning the budget for the installation of a DMP.

### Potential implications

Our empirical real-world study results can be used for further health and budget impact analyses as well as for decision analytic models assessing longer time horizons. Further research in health technology assessment should assess further aspects beyond effectiveness and cost-effectiveness such as the equity impact influenced by equal access for more patients if telemedicine is used in DMPs. Although we believe that this study shows robust results in favor of the DMP as a very cost-effective or even cost-saving option, future research could identify further predictors (i.e., effect modifiers) that allow a more individualized assessment of which patients benefit most and which subgroup offers the greatest cost savings.

## Conclusion

The economic evaluation along the cohort study showed that HerzMobil Tirol is very cost-effective compared to usual care based on the 1-year time horizon of the clinical cohort study. HerzMobil Tirol was cost-saving in a sensitivity analysis correcting for randomly occurring “non-HF related costs.” Further research is needed on the long-term assessment of benefits and costs beyond the study period using decision-analytic modeling.

### Supplementary Information

Below is the link to the electronic supplementary material.

Supplementary file1 (DOCX 15 KB)

Supplementary file2 (DOCX 19 KB)

Supplementary file3 (DOCX 13 KB)

Supplementary file4 (DOCX 64 KB)

## Data Availability

The data that support the findings of this study are available on request from the corresponding author.

## References

[1] Ambrosy AP et al (2014) The global health and economic burden of hospitalizations for heart failure: lessons learned from hospitalized heart failure registries. J Am Coll Cardiol 63:1123–1133 24491689 10.1016/j.jacc.2013.11.053

[2] Cook C, Cole G, Asaria P, Jabbour R, Francis DP (2014) The annual global economic burden of heart failure. Int J Cardiol 171:368–376 24398230 10.1016/j.ijcard.2013.12.028

[3] Benjamin EJ et al (2017) Heart disease and stroke statistics-2017 update: a report from the American Heart Association. Circulation 135:e146–e603 28122885 10.1161/CIR.0000000000000485 PMC5408160

[4] Crespo-Leiro MG et al (2016) European Society of Cardiology Heart Failure Long-Term Registry (ESC-HF-LT): 1-year follow-up outcomes and differences across regions. Eur J Heart Fail 18:613–625 27324686 10.1002/ejhf.566

[5] Dharmarajan K, Masoudi FA, Spertus JA, Li SX, Krumholz HM (2013) Contraindicated initiation of beta-blocker therapy in patients hospitalized for heart failure. JAMA Intern Med 173:1547–1549 23797379 10.1001/jamainternmed.2013.7717 PMC4043342

[6] Di Tanna GL et al (2019) Evaluating cost-effectiveness models for pharmacologic interventions in adults with heart failure: a systematic literature review. Pharmacoeconomics 37:359–389 30596210 10.1007/s40273-018-0755-x PMC6386015

[7] Blum MR et al (2020) Cost-effectiveness of transitional care services after hospitalization with heart failure. Ann Intern Med 172:248–257 31986526 10.7326/M19-1980

[8] Blum K, Gottlieb SS (2014) The effect of a randomized trial of home telemonitoring on medical costs, 30-day readmissions, mortality, and health-related quality of life in a cohort of community-dwelling heart failure patients. J Card Fail 20:513–521 24769270 10.1016/j.cardfail.2014.04.016

[9] Inglis SC, Clark RA, Dierckx R, Prieto-Merino D, Cleland JG (2015) Structured telephone support or non-invasive telemonitoring for patients with heart failure. Cochrane Database Syst Rev 2015:CD007228 26517969 10.1002/14651858.CD007228.pub3 PMC8482064

[10] Sydow H et al (2022) Cost-effectiveness of noninvasive telemedical interventional management in patients with heart failure: health economic analysis of the TIM-HF2 trial. Clin Res Cardiol 111:1231–1244 34894273 10.1007/s00392-021-01980-2 PMC9622523

[11] Poelzl G et al (2022) Feasibility and effectiveness of a multidimensional post-discharge disease management programme for heart failure patients in clinical practice: the HerzMobil Tirol programme. Clin Res Cardiol 111:294–307 34269863 10.1007/s00392-021-01912-0

[12] Von der Heidt A et al (2014) HerzMobil Tirol network: rationale for and design of a collaborative heart failure disease management program in Austria. Wien Klin Wochenschr 126:734–741 25392254 10.1007/s00508-014-0665-7

[13] Ramsey SD et al (2015) Cost-effectiveness analysis alongside clinical trials II-An ISPOR Good Research Practices Task Force report. Value Health 18:161–172 25773551 10.1016/j.jval.2015.02.001

[14] Hunink MM et al (2014) Decision making in health and medicine: integrating evidence and values. Textbook, Cambridge University Press. 10.1017/CBO9781139506779

[15] Neumann PJ, Sanders GD, Russell LB, Siegel JE, Ganiats TG (2016) Cost-effectiveness in health and medicine. Textbook, Oxford University Press

[16] Sjölander A, Greenland S (2013) Ignoring the matching variables in cohort studies - when is it valid and why? Stat Med 32:4696–4708 23761197 10.1002/sim.5879

[17] Johnson ML, Crown W, Martin BC, Dormuth CR, Siebert U (2009) Good research practices for comparative effectiveness research: analytic methods to improve causal inference from nonrandomized studies of treatment effects using secondary data sources: the ISPOR Good Research Practices for Retrospective Database Analysis Task Force Report-Part III. Value Health 12:1062–1073 19793071 10.1111/j.1524-4733.2009.00602.x

[18] Cox E et al (2009) Good research practices for comparative effectiveness research: approaches to mitigate bias and confounding in the design of nonrandomized studies of treatment effects using secondary data sources: the International Society for Pharmacoeconomics and Outcomes Research Good Research Practices for Retrospective Database Analysis Task Force Report-Part II. Value Health 12:1053–1061 19744292 10.1111/j.1524-4733.2009.00601.x

[19] Husereau D et al (2013) Consolidated Health Economic Evaluation Reporting Standards (CHEERS) statement. Value Health 16:e1-5 23538200 10.1016/j.jval.2013.02.010

[20] Husereau D et al (2022) Consolidated Health Economic Evaluation Reporting Standards 2022 (CHEERS 2022) statement: updated reporting guidance for health economic evaluations. Value Health 25:3–9 35031096 10.1016/j.jval.2021.11.1351

[21] Garrison LP, Towse A (2017) Value-based pricing and reimbursement in personalised healthcare: introduction to the basic health economics. J Pers Med 7:10 28869571 10.3390/jpm7030010 PMC5618156

[22] Neumann PJ, Cohen JT, Weinstein MC (2014) Updating cost-effectiveness–the curious resilience of the $50,000-per-QALY threshold. N Engl J Med 371:796–797 25162885 10.1056/NEJMp1405158

[23] Dierckx R, Inglis SC, Clark RA, Prieto-Merino D, Cleland JG (2017) Telemedicine in heart failure: new insights from the Cochrane meta-analyses. Eur J Heart Fail 19:304–306 28251777 10.1002/ejhf.759

[24] Koehler F et al (2018) Efficacy of telemedical interventional management in patients with heart failure (TIM-HF2): a randomised, controlled, parallel-group, unmasked trial. Lancet 392:1047–1057 30153985 10.1016/S0140-6736(18)31880-4

[25] Feltner C et al (2014) Transitional care interventions to prevent readmissions for persons with heart failure: a systematic review and meta-analysis. Ann Intern Med 160:774–784 24862840 10.7326/M14-0083

[26] Van Spall HGC et al (2017) Comparative effectiveness of transitional care services in patients discharged from the hospital with heart failure: a systematic review and network meta-analysis. Eur J Heart Fail 19:1427–1443 28233442 10.1002/ejhf.765

[27] Mai Ba H, Son YJ, Lee K, Kim BH (2020) Transitional care interventions for patients with heart failure: an integrative review. Int J Environ Res Public Health 17:2925 32340346 10.3390/ijerph17082925 PMC7215305

[28] Vestergaard AS, Hansen L, Sorensen SS, Jensen MB, Ehlers LH (2020) Is telehealthcare for heart failure patients cost-effective? An economic evaluation alongside the Danish TeleCare North heart failure trial. BMJ Open 10:e031670 31992604 10.1136/bmjopen-2019-031670 PMC7045102

[29] Sculpher MJ, Claxton K, Drummond M, McCabe C (2006) Whither trial-based economic evaluation for health care decision making? Health Econ 15:677–687 16491461 10.1002/hec.1093

[30] Siebert U (2003) When should decision-analytic modeling be used in the economic evaluation of health care? Eur J Health Econom formerly: HEPAC 4:143–150 10.1007/s10198-003-0205-2

