# Supplementary material for: Cost-effectiveness of a multidimensional post-discharge disease management program for heart failure patients—economic evaluation along a one-year observation period
Source: Clin Res Cardiol. 2024 Feb 14;113(8):1232–41. doi: 10.1007/s00392-024-02395-5 (PMC11269486; doi:10.1007/s00392-024-02395-5)
Supplement: Supplementary file 1 — Supplementary file1 (DOCX 15 KB) [file 392_2024_2395_MOESM1_ESM.docx]

Table S1 - Distribution of patients among the hospitals in Tyrol and tariffs for DRG point

| **Hospital Center** | **HerzMobil**  **(n=251)** | **Usual Care**  **(n=257)** | **EUR per DRG point 2022** |
| --- | --- | --- | --- |
| LKH Hall | 56 | 8 | 1.40 |
| LKH Hochzirl | 29 (LKH Natters/ Hochzirl) | 8 | 1.35 |
| LKH Innsbruck | 110 | 187 | 1.45 |
| LKH Natters | 29 (LKH Natters/Hochzirl) | 54 | 1.35 |
| BKH Schwaz | 40 | - | 1.15 |
| KH St.Vinzenz Zams | 13 | - | 1.20 |
| BKH Kufstein | 3 | - | 1.15 |

DRG: Diagnostic Related Group
