# Supplementary material for: Cost-effectiveness of a multidimensional post-discharge disease management program for heart failure patients—economic evaluation along a one-year observation period
Source: Clin Res Cardiol. 2024 Feb 14;113(8):1232–41. doi: 10.1007/s00392-024-02395-5 (PMC11269486; doi:10.1007/s00392-024-02395-5)
Supplement: Supplementary file 2 — Supplementary file2 (DOCX 19 KB) [file 392_2024_2395_MOESM2_ESM.docx]

Table S2 - Incremental cost-effectiveness base-case analysis additional health outcomes

|  | **Event-free-survival [days]** | **∆ Event-free-survival [days]]** | **Total Costs**  **[EUR]** | **∆Total Costs**  **[EUR]** | **ICER** |
| --- | --- | --- | --- | --- | --- |
| Usual Care | 4.69 | 0.54 | 6.943 | 523 | 969 |
| HerzMobil Tirol | 5.23 |  | 7.466 |  |  |
|  | **Life years (LY)** | **∆ LY** | **Total Costs**  **[EUR]** | **∆Total Costs**  **[EUR]** | **ICER** |
| Usual Care | 0,849 | 0.11 | 6.943 | 523 | 4,750 |
| HerzMobil Tirol | 0,959 |  | 7.466 |  |  |
|  | **Number of rehospitalization**  **within 1 year** | **Number of rehospitalization avoided within 1 year** | **Total Costs**  **[EUR]** | **∆Total Costs**  **[EUR]** | **ICER** |
| Usual Care | 0.59 | 0.12 | 6.943 | 523 | 4,358 |
| HerzMobil Tirol | 0.47 |  | 7.466 |  |  |

ICER: Incremental cost-effectiveness ratio
