# Supplementary material for: Cost-effectiveness of a multidimensional post-discharge disease management program for heart failure patients—economic evaluation along a one-year observation period
Source: Clin Res Cardiol. 2024 Feb 14;113(8):1232–41. doi: 10.1007/s00392-024-02395-5 (PMC11269486; doi:10.1007/s00392-024-02395-5)
Supplement: Supplementary file 3 — Supplementary file3 (DOCX 13 KB) [file 392_2024_2395_MOESM3_ESM.docx]

Table S3 Cost-effectiveness analysis HerzMobil Tirol vs. usual care - sensitivity analysis after exclusion of six patients with non-HF related costs

|  | **Hospital-free**  **survival [years]** | **Incremental Hospital-free**  **survival years** | **Total costs [EUR]** | **Incremental Total costs [EUR]** | **ICER**  **[EUR/HFLYG]** |
| --- | --- | --- | --- | --- | --- |
| Usual Care | 0.82 | 0.11 | 6,040 | -992 | HMT Dominant* |
| HerzMobil Tirol | 0.93 |  | 5,048 |  |  |

*Cost-saving; ICER: incremental cost-effectiveness ratio; HFLYG: hospital-free life years gained
