# Supplementary material for: Cost-effectiveness of a multidimensional post-discharge disease management program for heart failure patients—economic evaluation along a one-year observation period
Source: Clin Res Cardiol. 2024 Feb 14;113(8):1232–41. doi: 10.1007/s00392-024-02395-5 (PMC11269486; doi:10.1007/s00392-024-02395-5)
Supplement: Supplementary file 4 — Supplementary file4 (DOCX 64 KB) [file 392_2024_2395_MOESM4_ESM.docx]

**Figure S1** Cost-effectiveness plane HerzMobil Tirol vs. usual care for sensitivity analysis after exclusion of six patients with non-HF related costs


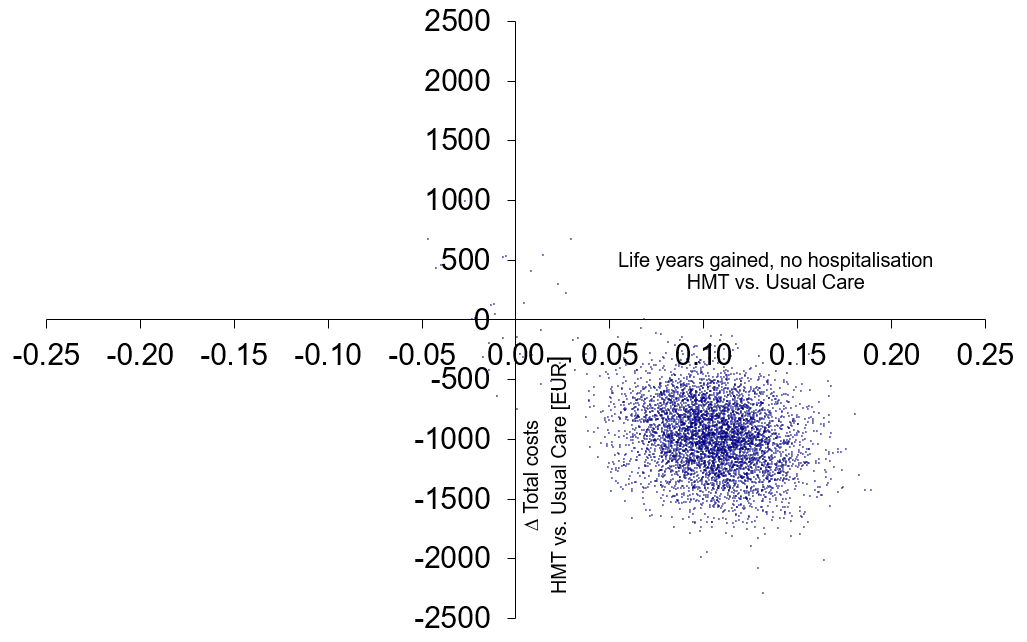


EUR: Euro; HMT: HerzMobil Tirol; DMP: disease management program

Health Outcome: Hospital-free survival in years

Cost Outcome: DMP + rehospitalization costs

Cost effectiveness with 5000 bootstrap simulations

mean life years gained HMT vs. Usual Care: 0.105 (95% confidence interval CI: 0.06 to 0.15), mean Δ total cost [EUR] HMT vs. Usual Care: -977.5 (95% CI: -1555.2 to -389.7)
